# Numerical Simulations of Directed Self-Assembly in Diblock Copolymer Films using Zone Annealing and Pattern Templating

**DOI:** 10.1038/s41598-017-05565-w

**Published:** 2017-07-12

**Authors:** Joseph D. Hill, Paul C. Millett

**Affiliations:** Department of Mechanical Engineering, University of Arkansas, Fayetteville, AR 72701 USA

## Abstract

Bulk fabrication of surface patterns with sub-20 nm feature sizes is immensely desirable for many existing and emerging technologies. Directed self-assembly (DSA) of block copolymers (BCPs) has been a recently demonstrated approach to achieve such feature resolution over large-scale areas with minimal defect populations. However, much work remains to understand and optimize DSA methods in order to move this field forward. This paper presents large-scale numerical simulations of zone annealing and chemo-epitaxy processing of BCP films to achieve long-range orientational order. The simulations utilize a Time-Dependent Ginzburg-Landau model and parallel processing to elucidate relationships between the magnitude and velocity of a moving thermal gradient and the resulting BCP domain orientations and defect densities. Additional simulations have been conducted to study to what degree orientational order can be further improved by combining zone annealing and chemo-epitaxy techniques. It is found that these two DSA methods do synergistically enhance long-range order with a particular relationship between thermal gradient velocity and chemical template spacing.

## Introduction

The ability to accurately produce nanoscale surface patterns has become a driving factor for research in many fields. As the need for nanoscale manufacturing increases, new processing technologies must be developed in order to meet industry needs. A number of different patterning methods have shown progress in decreasing feature size beyond the capabilities of traditional lithography^1^. These methods include extreme ultraviolet lithography, nanoimprinting, maskless lithography, and directed self-assembly (DSA) of block copolymers (BCP)^2–7^. BCPs can form very monodisperse periodic domains with domain sizes that can be tailored as small as 5 nm. The domain sizes in a microphase separated BCP matrix correspond with the molecular lengths of each section of the copolymer chain^8^. Linear BCPs are commonly used for patterning research and applications, although other chain architectures are also being explored^9–11^. The phase-separated morphologies of linear BCPs are dependent on the interaction parameter *χ*, degree of polymerization *N*, and the ratio of the length of each block *f*. Stable and meta-stable morphologies include ordered spheres, cylinders, lamellae, and gyroid structures^12–16^. The variety, regularity, and small size of these structures make BCPs highly attractive for many nano-manufacturing applications.

The microphase separation of BCP thin films has been broadly studied and shown to be useful for surface patterning and membrane applications^17^. These formations, while periodic, are prone to defects such as dislocations and grain boundaries that detract from their utility in some applications. A variety of strategies have been developed to extend defect-free uniformity during self-assembly including solvent annealing^18^, shear alignment^19^, epitaxy^20–23^, zone casting^24^, and field alignment^25–29^. Studies of domain morphologies have shown that directional quenching and/or annealing of a BCP film can considerably enhance the order within the system, whereby the orientation of the periodic structures is dependent on the direction and the velocity of a moving thermal gradient field^30^. This approach, termed zone annealing, involves passing a BCP film through a series of hot and cold temperature fields to impose a moving thermal gradient within the film.

The earliest implementation of zone annealing utilized temperatures in the hot zone above the order-disorder temperature (*T*_*ODT*_) of the BCP film^31^, now designated as hot zone annealing (HZA). Subsequent efforts applied lower temperatures in the hot zone in the range of *T*_*G*_ < *T* < *T*_*ODT*_ where *T*_*G*_ is the glass transition temperature, and this approach is designated cold zone annealing (CZA)^32–36^. In HZA, new microdomains form and become aligned in the cooling edge of the zone, as opposed to CZA whereby new microdomains form in a narrow range on the heating edge of the zone once the temperature exceeds *T*_*G*_. A somewhat related, yet distinct, approach known as zone casting^37^ involves injecting a BCP solution at an elevated temperature onto a colder substrate as the substrate is withdrawn perpendicular to the injection. The injection rate and withdrawal velocity are carefully controlled to maintain film thicknesses between 100 *nm* and a few micrometers. It has been observed that orientation of ordered lamellae are affected by the casting temperature and withdrawal rate. Remarkably, both CZA and zone casting methods have shown essentially defect-free microdomain patterns when the thermal gradient velocity (or, the withdrawal velocity for zone casting) is kept below ~5 *μ*m/s. Furthermore, the sharpness of the thermal gradient has been shown to affect the periodic domain orientation. A sharp thermal gradient (∇*T* ~ 45 K/mm)^33^ in CZA has been shown to produce vertical orientation (i.e. in the z-direction of the film) while broader thermal gradients (∇*T* ~ 17 K/mm) result in parallel alignment for similar velocities.

Computational studies including both particle-based and field-based simulations have expanded our understanding of many different DSA approaches to control BCP morphology^8, 35, 38–41^. In particular, mesoscopic field-based methods including self-consistent field theory and time-dependent Ginzburg-Landau (TDGL) models offer the required spatial resolution to represent the BCP domain structure while also allowing the computational efficiency to analyze fairly large sections of a BCP matrix. With regards to DSA, numerical simulations have been used to study BCP defect behavior in topologically and/or chemically patterned substrates^42, 43^, magnetic- and electric-field alignment with and without nanoparticle loading^44–46^, as well as a few studies of thermal gradient zone annealing alignment^30, 35, 47, 48^.

In this work, we present large-scale TDGL simulations to analyze the degree of BCP microdomain orientation and the defect populations during the CZA process. We systematically vary both the magnitude and the velocity of a moving thermal gradient translating across an initially disordered BCP film. Results illustrate direct relationships between these two parameters and the resultant microdomain orientations that emerge. Furthermore, we investigate how a combination of CZA and chemical template alignment patterns can synergistically enhance each other to further improve long-range order within a film. The addition of templating patterns effectively allows increased CZA velocities while maintaining defect-free domains.

## Methods

In the current work, we consider an idealized AB diblock copolymer film with equal volume fractions of the A and B monomers, *ϕ*_*A*_ and *ϕ*_*B*_. We strive to replicate CZA experiments^32^, in which a film is spin-coated as a dense, disordered layer that is largely devoid of a solvent. Hence, this binary system – if assumed to be incompressible – should reasonably obey the condition *ϕ*_*A*_ + *ϕ*_*B*_ = 1, and we therefore can consider one independent volume fraction, e.g. *ϕ*_*A*_. We utilize the coarse-grained TDGL model commonly invoked to simulate relatively large regions of a block copolymer phase-separated microstructure^37, 49^. A Cahn-Hilliard equation describes the kinetic morphology change by spatially and temporally updating an order parameter, here corresponding to *ϕ*_*A*_:

1 $$\frac{\partial {\varphi }_{A}}{\partial t}=\nabla \cdot ({M}_{A}\nabla \frac{\delta F({\varphi }_{A})}{\delta {\varphi }_{A}})+\xi ({\bf{r}},t),$$

where *M*_*A*_ represents the mobility of monomer A (assumed to be equal to *M*_*B*_), *F*(*ϕ*_*A*_) is a free energy functional representing the short- and long-range chemical energy of mixing between the monomer species, and *ξ*(**r**, *t*) is a normalized random noise term spatially averaging zero. Equation (1) ensures global conservation of *ϕ*_*A*_.

Micro-phase separation is induced by the proper definition of *F*(*ϕ*_*A*_), which consists of both short- and long-range terms:

2 $$F({\varphi }_{A})={F}_{S}({\varphi }_{A})+{F}_{L}({\varphi }_{A}),$$

where the short-range chemical mixing energy is defined by a simple polynomial expression combined with a gradient term:

3 $${F}_{S}({\varphi }_{A})=\int [\psi {\varphi }_{A}^{2}{\mathrm{(1}-{\varphi }_{A})}^{2}+\kappa {|\nabla {\varphi }_{A}|}^{2}]d{\bf{r}},$$

where the first term represents a double-well curve that produces a miscibility gap by penalizing volume fractions between *ϕ*_*A*_ = 0 and *ϕ*_*A*_ = 1. A polynomial expression such as this is routinely substituted for a Flory-Huggins model^50^, as it provides better numerical efficiency while still qualitatively describing an immiscible mixture. The second term penalizes gradients in *ϕ*_*A*_ that occur at A-B interfaces. As is typical in Cahn-Hilliard models, the A-B interfacial energy is proportional to $\sqrt{\psi \kappa }$ which is therefore also proportional to the Flory-Huggins factor *χN*. The simulations presented here represent BCPs in the strong segregation regime. The long-range term penalizes domain growth due to the physical attachment of the A and B chains, and is defined by:

4 $${F}_{L}({\varphi }_{A})=\frac{\alpha }{2}\int d{\bf{r}}\int d{\bf{r}}{\boldsymbol{^{\prime} }}G({\bf{r}}-{\bf{r}}{\boldsymbol{^{\prime} }}){\varphi }_{A}({\bf{r}}){\varphi }_{A}({\bf{r}}{\boldsymbol{^{\prime} }})$$

where the Green function *G*(**r** − **r**′) satisfies ∇^2^ *G*(**r** − **r**′) = −*δ*(**r** − **r**′) and the coefficient *α* is proportional to (*Nf*(1 − *f*))^−2^ where *N* is the total degree of polymerization and *f* is the fraction of A monomers on the chain. For all simulations herein, we assign *f* = 0.5 corresponding to lamellae morphologies on the BCP phase diagram. While this form of the Green function was developed to describe bulk BCP systems, it has been shown that significant deviation is not introduced near solid walls^51^.

**Figure 1:**
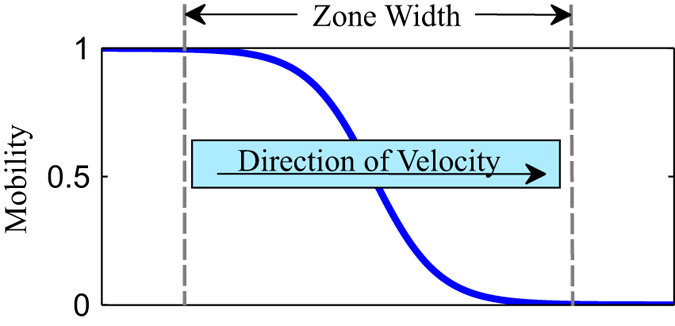
Simulations of zone annealing are performed with a spatiotemporal block copolymer mobility expressed in Eq. (5). As the thermal zone moves across the sample, the local mobility increases from values of zero to one.

It is known that the Flory-Huggins parameter *χ* exhibits an inverse dependence on temperature, which could be incorporated in the simulation model. However, because CZA experiments maintain temperatures well below *T*_*c*_, variations in *χ* are relatively unimportant compared with the highly temperature-dependent polymer mobility, particularly as the system transitions from below to above the glass transition temperature^35^. Therefore, in our simulations, the directional annealing front is modeled by prescribing a spatially- and temporally-dependent species mobility, *M*_*A*_. Shown in Fig. 1, we utilize a smooth functional form:

5 $${M}_{A}=\frac{1}{2}[1-tanh(\frac{\mathrm{6(}{x}_{i}-{x}_{f})}{{w}_{zone}})]$$

where *x*_*i*_ is any x-position in the domain, *w*_*zone*_ is the width of the temperature transition zone, and *x*_*f*_ = *t* · Δ*t* · *v*_*zone*_ − 0.5 *w*_*zone*_ is the time-dependent x-position of the temperature transition zone (with *t*, Δ*t*, and *v*_*zone*_ being the current time step, the time step size, and the zone velocity in the x-direction, respectively). The results presented below use a variety of values for *v*_*zone*_ and *w*_*zone*_. For simulations of isothermal annealing, the mobility has a constant value of *M*_*A*_ = 1.

We furthermore investigate how chemoepitaxial templating and CZA together may synergistically enhance long-range order in block copolymer films. We introduce template stripes in the domains with a chemically favorable interaction with the B monomer, in a similar spirit to previous studies^51, 52^. It should be noted that the preferential strength of chemical templating is key to the efficiency of chemo-epitaxy. For this work, templating is enforced for a very narrow stripe, much smaller than the lamellar period, such that attraction of polymer B is induced in templated areas. This is modeled with a simple alteration of the short-range free energy functional that transforms a double-well curve into a single-well curve:

6 $${F}_{S}({\varphi }_{A})=\psi [{\varphi }_{A}^{2}{\mathrm{(1}-{\varphi }_{A})}^{2}+\eta {\varphi }_{A}^{2}]+\kappa {|{\rm{\Delta }}{\varphi }_{A}|}^{2}$$

where *η* is a field variable that represents the locations of epitaxial alignment patterns (*η* = 1 inside a stripe pattern and *η* = 0 elsewhere). This function encourages B-monomers at the stripe patterns, with an energetic penalty of placing an A-monomer on a stripe that is equivalent to placing an A-monomer in a B-block region in the bulk BCP matrix.

We numerically solve Eq. (1) with a straightforward explicit finite difference scheme using a forward Euler time derivative and central difference approximations for spatial derivatives. The calculations utilized reduced units of length ($\tilde{l}$) and time ($\tilde{t}$). All simulations were conducted with two-dimensional grids with a uniform grid spacing of Δ*x* = Δ*y* = 1 $\tilde{l}$. The time step size was kept fixed at Δ*t* = 0.03 $\tilde{t}$. The dimensions of the grids vary somewhat: for untemplated simulations a grid size of 2000 × 1000 was used, whereas for simulations with epitaxial stripe spacings of *L* ^*T*^ = 6 *L*_0_ and *L* ^*T*^ = 7 *L*_0_ grids of 1980 × 1020 and 2030 × 980 were used, respectively. Here, *L*_0_ is the lamellar period length for the block copolymer measured to be *L*_0_ = 10 $\tilde{l}$ for energy parameters of *ψ* = 1, *κ* = 1, and *α* = 0.1. Periodic boundary conditions are used in the y-direction and no-flux boundary conditions are enforced in the x-direction. Furthermore, the walls on the x-direction boundaries are chemically neutral with regards to the A- and B-blocks of the copolymer. Multiple zone velocities and zone widths were simulated, and the data points for each condition represent averages of 10 simulations with error bars indicating the standard error of the mean. Simulations are initialized independently as homogeneous solutions with small, random fluctuations in concentration about the mean. The simulations were executed with parallel computing to enable large grid sizes and the large parameter space explored. Due to the nature of our 2-dimensional domains, the simulations presented here are effectively portraying a mono-layer thin-film which precludes the inclusion of out-of-plane defects and treats chemical striping as a through-thickness alignment field as opposed to a local alignment field at the lower surface of the polymer melt.

**Figure 2:**
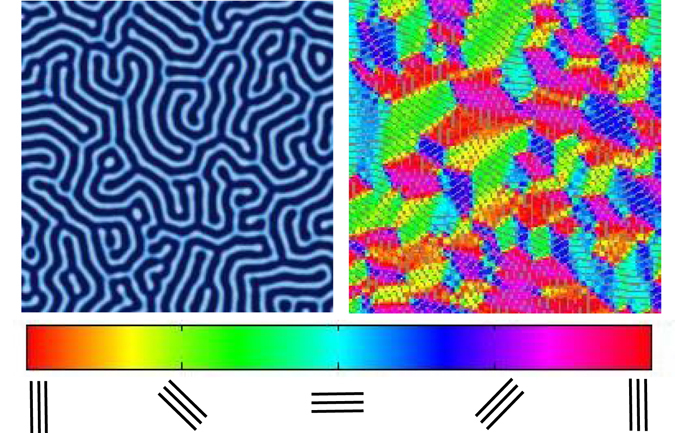
False-color imaging is used to show local lamellae orientation. The image here is obtained from an isothermally annealed simulation.

In order to analyze how the zone velocity affects the final orientation of the BCP microdomains, we run each sample through a false color orientation algorithm that calculates the orientation of each sample between 0 and 180 degrees, which can be easily visualized as shown in Fig. 2. Orientation directions at grid points within the discretized system are calculated based on the gradient of *ϕ*_*A*_ at each point. The percent orientation of each sample to within ± 30 degrees of parallel and ±30 degrees of perpendicular to the direction of the zone velocity (i.e. the *x*-direction) is then calculated.

## Results

### CZA Simulation Results

**Figure 3:**
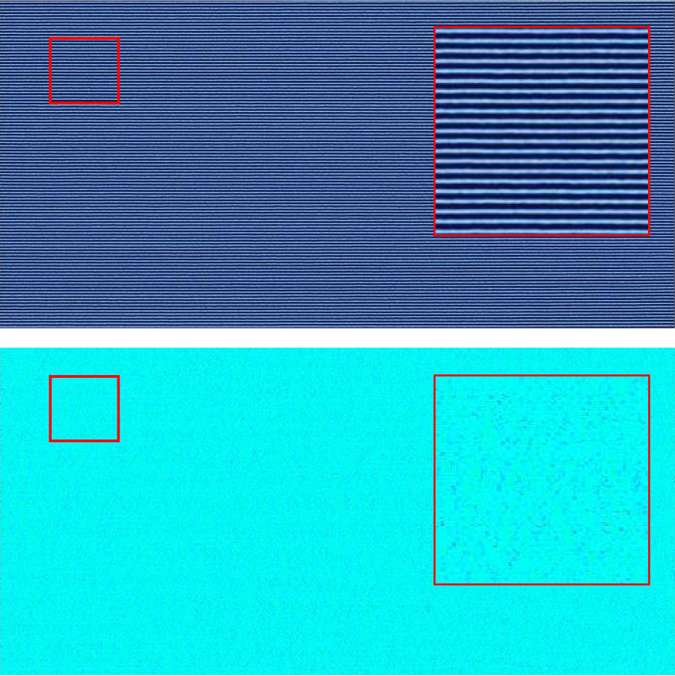
Snapshots of horizontally aligned lamellae obtained with a zone velocity of *v*_*zone*_ = 0.0233 and a zone width of *w*_*zone*_ = 80 $\tilde{l}$ without templating. The inhomogeneous mobility field moves from left to right across the domain – this image corresponds to the end of the simulation when the zone has moved across the entire sample. This sample contains no topological defects.

**Figure 4:**
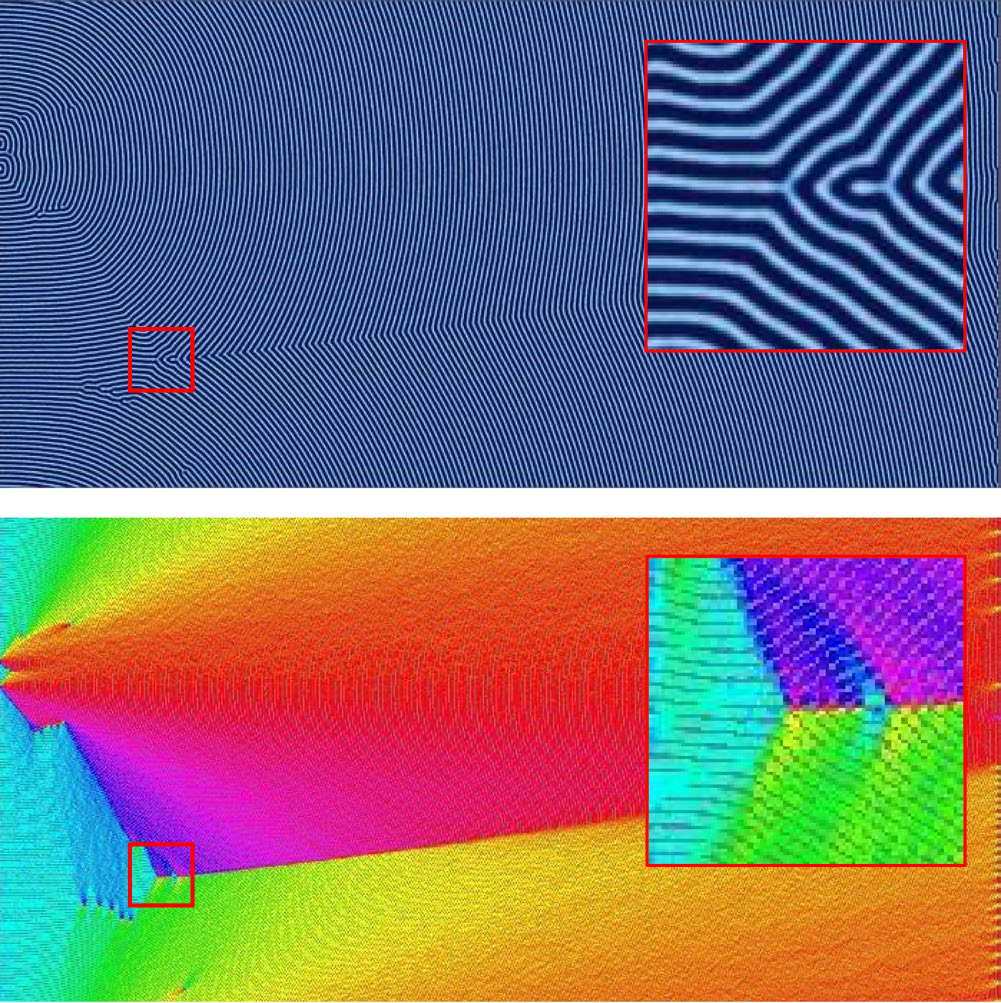
Same as Fig. 3 with a higher zone velocity of *v*_*zone*_ = 0.0645. Extended defects similar to grain boundaries emerge and generally extend from left to right in the direction of the zone translation.

**Figure 5:**
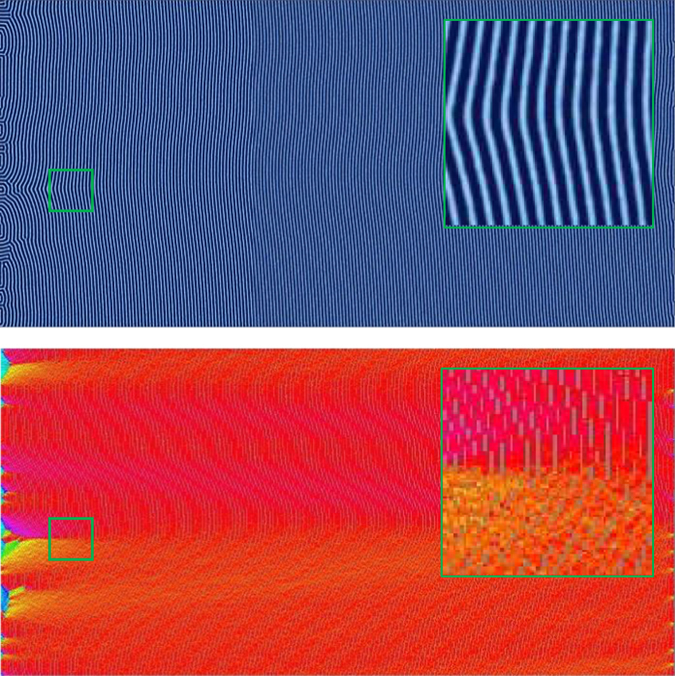
Same as Fig. 3 with a zone velocity of *v*_*zone*_ = 0.2301. A predominately perpendicular alignment has formed with the minor exception of the left boundary where the thermal zone enters the domain.

**Figure 6:**
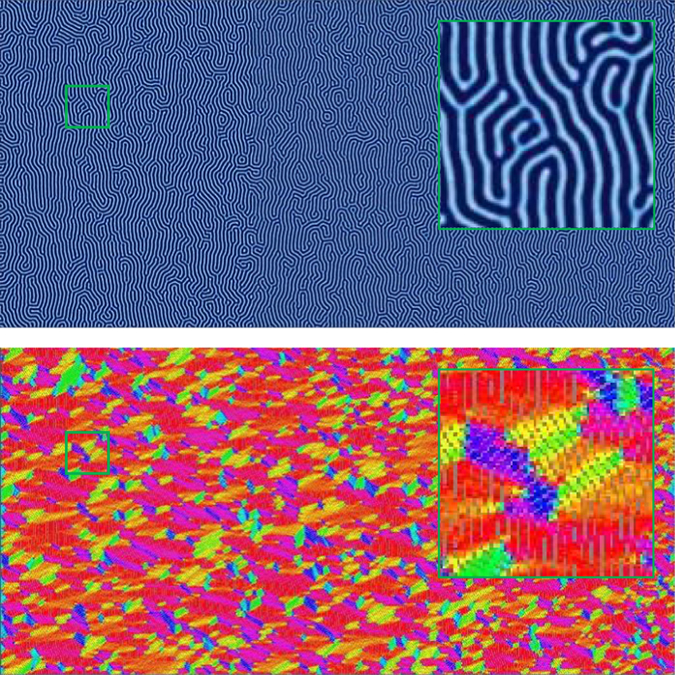
Same as Fig. 3 with a zone velocity of *v*_*zone*_ = 0.6366. Although the system contains some preference for perpendicular alignment, a significant quantity of defects exists at this velocity.

**Figure 7:**
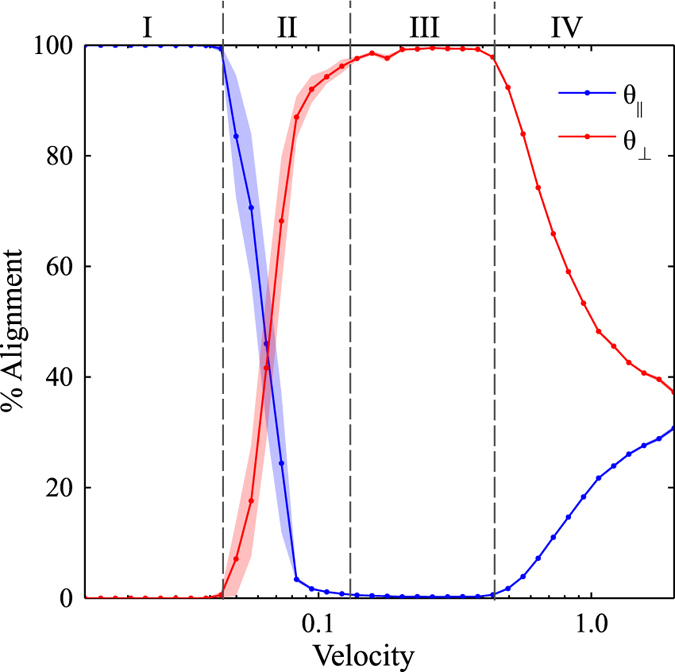
The percent alignment of lamellae to within ± 30 degrees of the directions parallel and perpendicular to the direction of zone velocity. The zone width is *w*_*zone*_ = 80 $\tilde{l}$. Four distinct regions emerge as a function of zone velocity: (I) parallel orientation, (II) transition from parallel to perpendicular orientation, (III) perpendicular orientation, and (IV) transition to unaligned orientations.

Characteristic results for zone velocities in the range of *v*_*zone*_ = 0.01–2.0 (with units of $\tilde{l}/\tilde{t}$ which will hereto forth be omitted) and a fixed zone width *w*_*zone*_ = 80 $\tilde{l}$ are shown in Figs 3, 4, 5 and 6 with the corresponding false color orientation fields. Orientation analysis of the simulation results shows behavior trends of the system as a function of zone velocity. Orientation percentages are plotted in Fig. 7 versus zone velocity, where velocity is plotted on a logarithmic scale. Considering the regions on the plot where one orientation (either parallel or perpendicular) is above 97% alignment (see Supplemental Information – Section 1 for additional details), four distinct regions present themselves within the data. The mean orientation values are plotted and the standard error of the mean is represented by the shaded areas around each line.

Region I is found at low velocities (see Fig. 7). In this region, the lamellae microdomains show a strong tendency to orient themselves parallel to the direction of the zone velocity. Simulation results in this region show little to no defects with minimal variability in the resultant structures. This parallel alignment was observed in CZA experiments at low zone velocities^32^. It is expected that further decreasing the zone velocity below the lower bound of our range will not produce noticeably different results. From a processing perspective, decreasing zone velocity is undesirable as it will increase annealing times. As zone velocity increases, we observe an interesting transition, whereby the BCP orientations shift from a parallel alignment to a perpendicular alignment with respect to the zone annealing direction. We designate this as Region II. In Region II, the transition appears to be steady on average, however with some variability as evidenced by the width of the error shading. With further increased zone velocity, the BCP alignment transitions to a nearly 100% perpendicular orientation, designated as Region III. For zone velocities in this region, it is only the far left boundary of the computational domain, where the temperature zone first enters the domain, that exhibits some variation in alignment (see Fig. 5). Near this boundary, which has neutral chemical preference, the BCP domains align either parallel or perpendicular to the boundary (Cong *et al*.^47^ analyzed wall interactions to a greater extent). However, in a very short distance, the BCP alignment becomes uniformly perpendicular as can be seen in the orientation plot of Fig. 5. With further increases in the zone velocity, the system exhibits decreased global alignment, as shown in Region IV, where the percent perpendicular and percent parallel alignment curves both approach 33%, which corresponds to their portion of the 180 degree range. In this region, the thermal front of the zone moves across the sample rapidly, and the results converge to those associated with isothermal annealing, and therefore the zone annealing process becomes ineffective as a directed self-assembly method.

The surprising result in Fig. 7 is the range of velocities associated with Region III whereby a perpendicular alignment is strongly favored. This region was not directly reported in the CZA experiments of Berry *et al*.^32^, although in personal correspondence Berry stated that some CZA experiments did result in a predominantly perpendicular alignment. On the other hand, the computational studies of Zhang *et al*.^30^, Bosse *et al*.^35^, and Cong *et al*.^47^ all observed parallel as well as perpendicular alignment depending on the zone velocity. However, those studies only chose a limited number of velocities and a general trend was not obtained. The zone-casting experiments of Tang *et al*.^37^ also revealed a tendency for either parallel or perpendicular lamella alignment depending on the casting temperature. Interestingly, this phenomenon of phase alignment during directional phase separation is perhaps broader than BCP systems. In particular, theoretical^53^ and experimental^54^ studies show that binary polymer mixtures undergoing directional phase separation also exhibit phase alignment either parallel or perpendicular to direction of the moving thermal gradient. Furukawa^53^ has proposed a convincing explanation stating that the concentration fluctuations in the early stage of phase separation may (or may not) be matched by the velocity of the thermal gradient that can lead to either parallel or perpendicular domains. It is remarkable that the patterns observed by Furukawa are so similar to the patterns observed here despite the fact that he simulated polymer-polymer phase separation (rather than BCP microphase separation) and he induced a moving quench boundary whereas we induce a moving mobility/annealing boundary. In addition, Komura *et al*.^55^ and Paquette^56^ studied the propagation velocities of order-disorder interface fronts in lamellar-phase BCPs using a similar numerical model as implemented here. Komura *et al*.^55^ found that for lamellae oriented perpendicular to the front direction, the front velocity varied between 0.005 and 0.03 (reduced units, defined as is here), a range very similar to our results in Fig. 7. This seems to indicate that a potential synchronization of zone velocity and the natural propagation velocity of perpendicular lamellae will favor this orientation.

**Figure 8:**
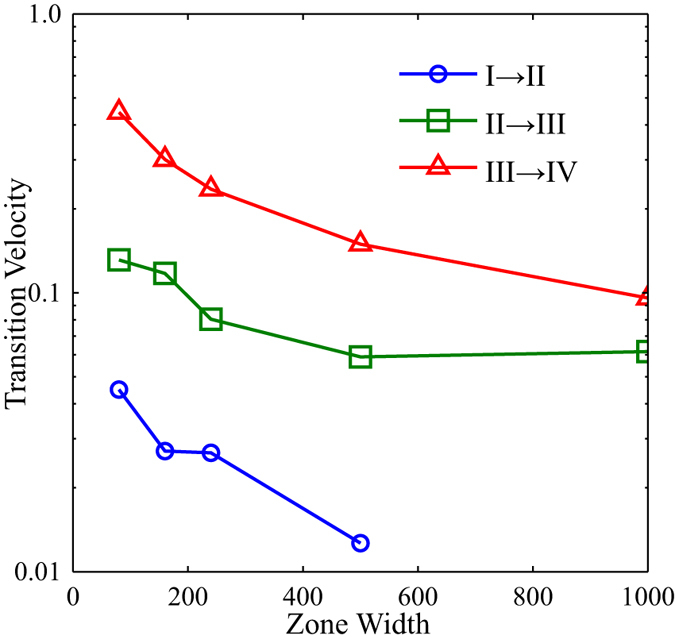
Transition velocities for Regions I-II, II-III, and III-IV as a function of the zone width, *w*_*zone*_. The full data and a description of the approach to calculate these transitions is provided in the Supplemental Information.

It is necessary to consider how the zone width (and therefore the thermal gradient) plays a role in the alignment of the BCP system. For data shown in Fig. 7, a single zone width (*w*_*zone*_ = 80 $\tilde{l}$ = 8 *L*_0_) was used. In comparison to experimental testing, this represents a very sharp, perhaps unrealistic, thermal gradient. Taking this into consideration, simulations were repeated using larger zone widths of *w*_*zone*_ = 160, 240, 500, and 1000 $\tilde{l}$ in order to verify that the same trends appear with larger zone widths. Results for five simulations at each velocity are averaged and the transition velocities are compared in Fig. 8 (see Supplemental Information – Section 2 for additional details). For each of the zone widths explored, the same trend can be seen where increasing zone velocity induces a transition from parallel alignment to perpendicular alignment and then a transition to zero alignment. Increased zone width also necessitates longer simulation times as the simulations begin and end with the thermal zone entirely outside of the simulated sample. As seen in Fig. 8, as zone width increases, transition velocities decrease slightly; however, in all cases the system exhibited parallel orientations at low velocities, perpendicular orientations at intermediate velocities, and unaligned orientations for high velocities.

### Templated CZA Simulation Results

In order to explore the possible synergy between CZA and chemical templating, we performed additional simulations that combine the effects of both techniques to determine the extent to which templating will allow increases in the zone velocity while maintaining a negligible quantity of defects. A similar analysis was recently conducted by Wan *et al*.^48^, however here we consider chemical templating in two directions independently. A range of zone velocities were tested in systems that contained either horizontally- or vertically-aligned straight-line chemical templates with template spacing ranging between 5–7 lamellar periods (or 50–70 $\tilde{l}$). Horizontal template stripes are parallel to the zone velocity, and their spacing is designated as *L*_||_, whereas vertical template spacing is designated as *L*_⊥_. Data was collected by averaging five simulations at each of the twenty-five zone velocities tested. For simplicity, the domain size at each template spacing was adjusted to fit both horizontal and vertical template patterns (i.e., the grid size must be an integer multiple of the template spacing).

**Figure 9:**
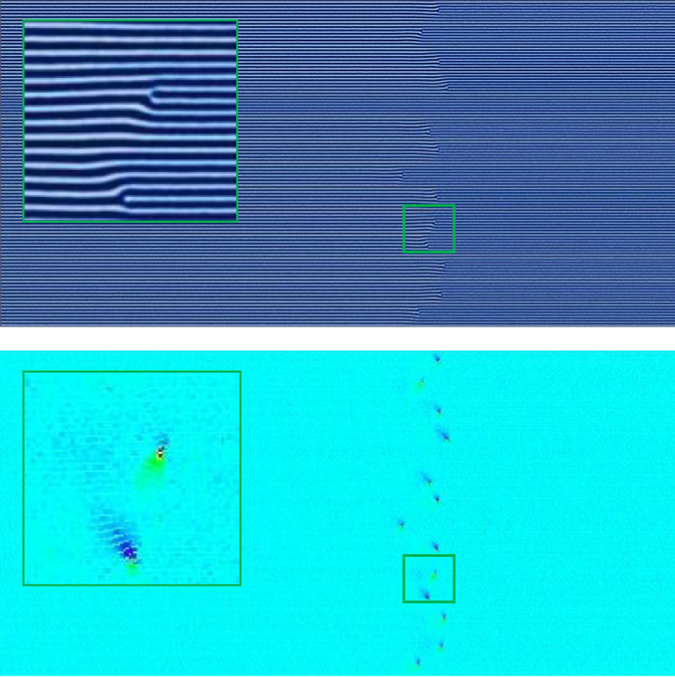
Simulation results for CZA with horizontal templating with a template spacing of *L*_||_ = 7 *L*_*o*_ at a zone velocity of *v*_*zone*_ = 0.103. At this velocity, the alignment is predominantly parallel with a few dislocation-type defects.

**Figure 10:**
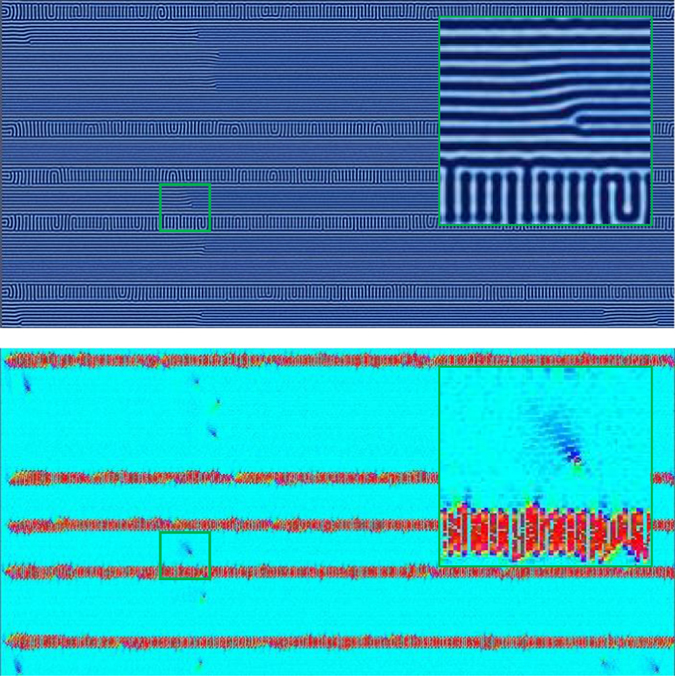
Same as Fig. 9 but with a zone velocity of *v*_*zone*_ = 0.215. At this velocity, the lamellae prefer a perpendicular orientation, which is restricted somewhat by the template pattern. A few bands of perpendicular lamellae develop between the template stripes.

**Figure 11:**
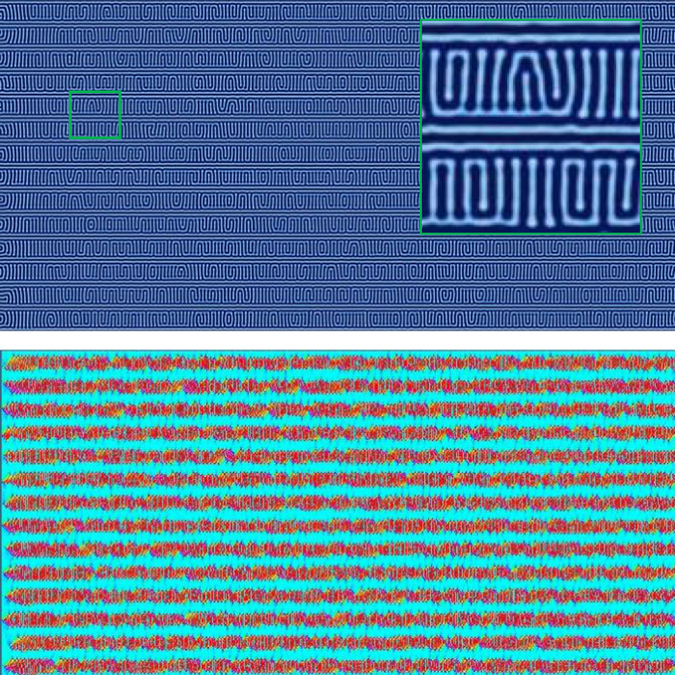
Same as Fig. 9 but with a zone velocity of *v*_*zone*_ = 0.310. At this velocity, the lamellae strongly prefer a perpendicular orientation, and all regions between the horizontal template patterns develop perpendicular lamellae bounded by parallel lamellae.

**Figure 12:**
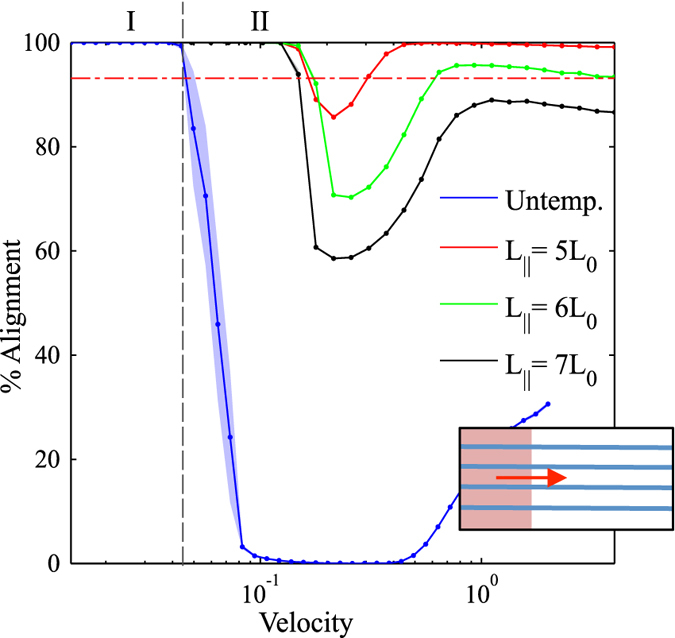
The percent alignment to within ±30 degrees of the direction parallel to the zone velocity for samples with parallel (horizontal) template stripes. The zone width is *w*_*zone*_ = 80 $\tilde{l}$. A shift in the transition velocity from Regions I to II is observed due to the presence of the template stripes. For comparison, the horizontal line shows average alignment of isothermally annealed samples with templating at *L*_||_ = 5*L*_0_ with annealing times equivalent to lowest velocity annealing times. Inset: template alignment (blue) is parallel to zone velocity (red).

For both horizontal and vertical templating patterns, we observed that the BCP morphology between adjacent template stripes was relatively unaffected by the morphology in the adjacent region. For domains with horizontal templating (i.e., parallel to the direction of the zone velocity), each of the regions of BCP orientation (i.e., Regions I, II, III, and IV) shift to higher velocities for each template spacing considered. In other words, faster velocities could be used to achieve the same percent alignment. Sample images of morphology from Regions I through III are shown respectively in Figs 9–11 for a template spacing of *L*_||_ = 7 *L*_0_. In Fig. 9, the zone velocity is *v*_*zone*_ = 0.103, which in the untemplated simulations corresponded with Region III, however here the alignment is predominately parallel, although with several dislocation-type defects. When *v*_*zone*_ is increased to 0.215 (Fig. 10), the system enters a regime where the BCP formation strongly favors perpendicular alignment, but is frustrated by the parallel template pattern. We observe certain regions between template stripes forming perpendicular lamellae and others forming parallel lamellae. These horizontal ‘bands’ do not seem to be affected by neighboring ‘bands’. These results are similar to those shown experimentally by Berry *et al*.^57^ where periodic templating aligned parallel to zone velocity via graphoepitaxy is used to affect orientation. A further increase in velocity to *v*_*zone*_ = 0.310 (Fig. 11) results in all regions between template stripes forming perpendicular alignment, separated by parallel lamellae directly overlapping the template stripes. Figure 12 plots the percent alignment data versus zone velocity for various values of *L*_||_. Of note, there is an increase in the velocity associated with a transition from parallel to perpendicular alignment.

**Figure 13:**
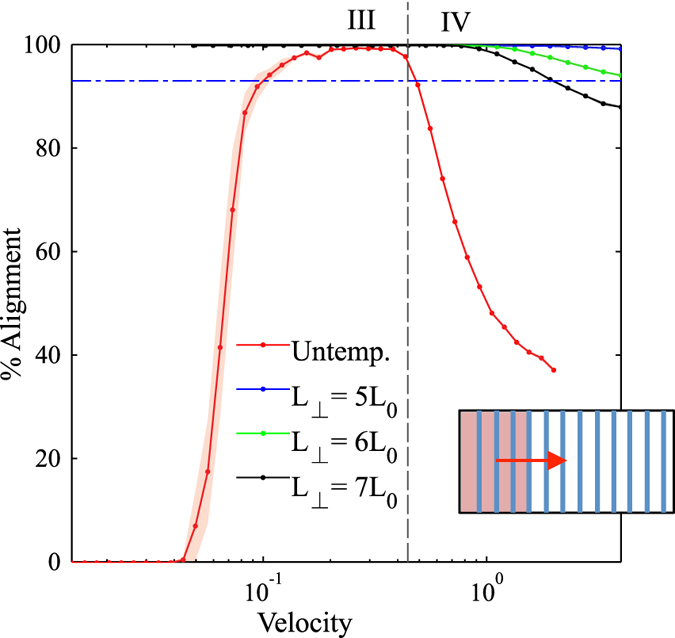
The percent alignment to within ±30 degrees of the direction perpendicular to the zone velocity for samples with perpendicular (vertical) template stripes. The zone width is *w*_*zone*_ = 80 $\tilde{l}$. Perpendicular template stripes provide the greatest shift in zone velocities for defect-free lamellae morphologies. For comparison, the horizontal line shows average alignment of isothermally annealed samples with templating at *L*_||_ = 5*L*_0_ with annealing times equivalent to lowest velocity annealing times. Inset: template alignment (blue) is perpendicular to zone velocity (red).

Due to the observation that untemplated zone annealing favors perpendicular alignment over parallel alignment at intermediate-to-high velocities, it seems plausible that perhaps a perpendicular template pattern may be best for enabling increased zone velocities. We executed the same set of simulations with a perpendicular template pattern, which starts at the left boundary of the domain, where the temperature front enters the domain. As a result of this positioning, perpendicular orientation is strongly encouraged near the leading edge and the parallel orientations associated with Regions I and II do not emerge within the range of velocities tested (i.e., even with very low velocities, the alignment was perpendicular). Figure 13 shows the percent alignment for various values of perpendicular template spacing (*L*_⊥_ = 5, 6, and 7 *L*_0_). Region III appears to have its upper and lower limits extended to notably higher velocities, showing a larger range of velocities where perpendicular alignment occurs. The transition from Region III to Region IV occurs at the highest velocities for all cases tested here, with transition velocities exceeding *v*_*zone*_ = 1.0 which is several times higher than untemplated results. This therefore suggests that templating with periodic stripes oriented perpendicular to the direction of zone velocity is optimal for enhancing CZA alignment of BCP films. For comparison, Figs 12 and 13 show the average orientation for isothermally annealed samples with template spacing of *L*_||_ = 5 *L*_0_. Annealing times for these samples are equivalent to the annealing time of lowest velocity samples annealed using CZA. Longer isothermal annealing times are expected to yield better alignment. While it is outside the scope of this work, it should be noted that for large samples, isothermal annealing may yield equivalent results with equivalent processing times as CZA times are dependent on the zone velocity and dimension of the sample in the direction of zone annealing.

## Conclusions

Large-scale numerical simulations have been performed to improve our understanding of CZA as a directed self-assembly strategy for BCP thin films. The orientation of lamellae is found to be highly dependent on the zone velocity, with subsequent transitions from parallel to perpendicular to unaligned orientations with increasing velocity. The width of the temperature zone, associated with the thermal gradient of the zone, was also studied and found to only slightly shift the transition regions, however with no qualitative change in the observed morphologies. We do point out that even our largest zone width is likely an order of magnitude below those formed in CZA experiments.

The combination of CZA and chemo-epitaxy can extend the range of velocities at which long-range orientational order can be achieved, a result that has implications for the efficient processing of nanopatterned surfaces. Here, we investigated linear stripe chemical template patterns and found that template stripes oriented perpendicular to the zone velocity resulted in the greatest benefit for improving orientational order. This template orientation takes advantage of the natural tendency of the BCP microdomains to orient perpendicular to the zone velocity at intermediate-to-high velocities. We note that the two-dimensional domains employed in our simulations allowed larger sample regions, however, they prohibited the possibility of out-of-plane microdomain alignment. The results of this study enable the first large-scale analysis of the transition of lamellae orientations from parallel to perpendicular alignment during zone annealing with a wide range of zone velocities and widths, and reveal that particularly applying perpendicular chemoepitaxial template stripes allow improved alignment compared with parallel templating.

## Electronic supplementary material

Supplementary Info
