# Supplementary material for: Numerical Simulations of Directed Self-Assembly in Diblock Copolymer Films using Zone Annealing and Pattern Templating
Source: Sci Rep. 2017 Jul 12;7:5250. doi: 10.1038/s41598-017-05565-w (PMC5507907; doi:10.1038/s41598-017-05565-w)
Supplement: Supplementary file 1 — Supplementary Info [file 41598_2017_5565_MOESM1_ESM.pdf]

Supplementary Information:
Numerical Simulations of Directed Self-Assembly in Diblock Copolymer Films using Zone Annealing and Pattern Templating

Joseph D. Hill[1] and Paul C. Millett[1*]

[1]Department of Mechanical Engineering, University of Arkansas, Fayetteville, AR 72701
[*]pmillett@uark.edu

Section 1. Region Assessment

The transitions between regions are determined by considering the velocities where mean data crosses a horizontal line drawn at 97% orientation via linear interpolation between data points. In transitions from Region I to Region II and Region III to Region IV where the mean orientation transitions from above to below the threshold, the earliest transitions are selected to determine region boundaries where multiple transitions occur as a result of fluctuations in the data. In transitions from Region II to Region III, the last transition is selected. This method of determining boundaries minimizes the areas of Regions I and III and ensures that they only contain velocities where the mean is above the required threshold. Figure S1 depicts the transition velocities for data collected using a zone width of 160 $\tilde{l}$ where both instances described above occur. Shaded areas on Fig. S1 represent the standard error of the mean for the corresponding orientation data.

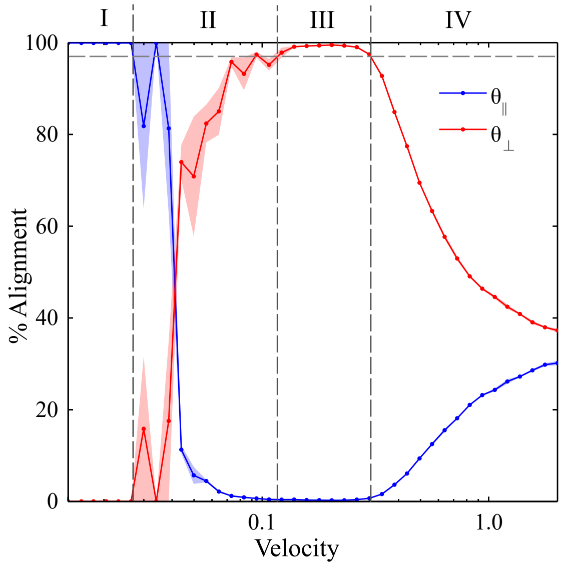

Figure S1. Orientation data for w_{zone} = 160 $\tilde{l}$ with untemplated domains. The threshold alignment and transition velocities are marked with dashed lines.

Section 2. Transition Velocity as a Function of Zone Width

Figures S2 and S3 show parallel and perpendicular orientation data, respectively, for each zone width considered in this work, for the untemplated simulations. The alignment threshold (97% alignment) is marked with a horizontal dashed line. Vertical dashed lines corresponding to the same color of the data depict calculated transition velocities. In Fig. S2, arrows depict the area corresponding to Region I (note that for w_{zone} = 1000 $\tilde{l}$, Region I does not present for the range of velocities simulated). In Fig. S3, double-headed arrows indicate the range of velocities associated with Region III. Each of these calculated boundary values are shown in Fig. 8 of the article.

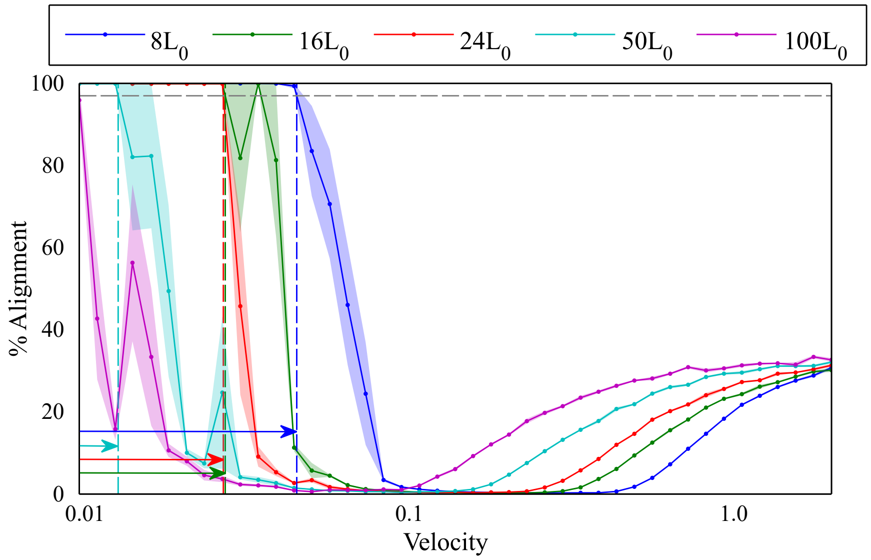

Figure S2. Parallel orientation data for tested values of w_{zone}. Dashed lines depict threshold alignment (grey) and transition velocities (colors correspond to w_{zone} data). Region I is depicted with arrows.

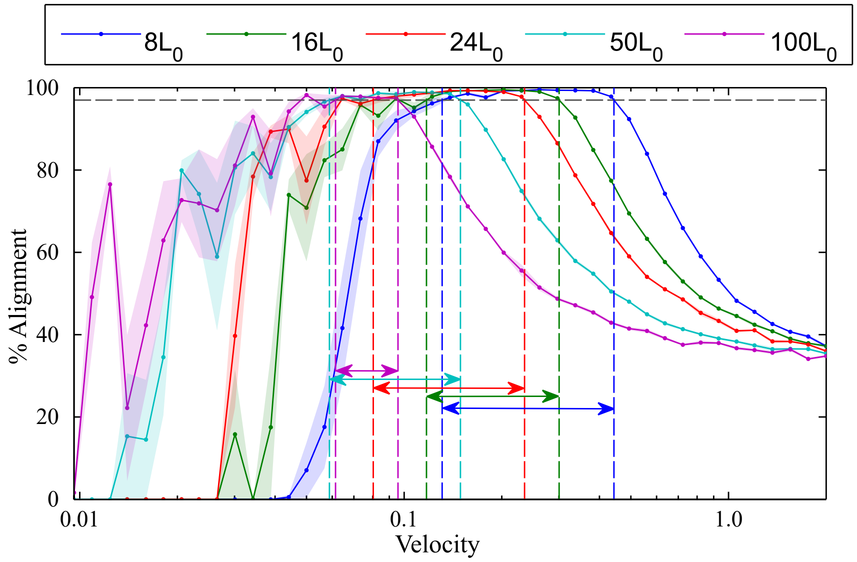

Figure S3. Perpendicular orientation data for tested values of w_{zone}. Dashed lines depict threshold alignment (grey) and transition velocities (colors correspond to w_{zone} data). Region III is depicted with double-headed arrows.
